# Estimation of the latent therapeutic demand for albumin in the USA: a focus on three indications

**DOI:** 10.1186/cc14434

**Published:** 2015-03-16

**Authors:** A Farrugia, M Bansal

**Affiliations:** 1 University of Western Australia, Perth, Australia; 2 Thought Semantics LLC, Sterling, VA, USA

## Introduction

The use of albumin in therapeutics is controversial in several areas and requires assessment based on evidence for effective resource allocation. Supported indications include sepsis, areas of hepatic diseases and coronary artery bypass grafts (CABG). Latent therapeutic demand (LTD) [1] is the underlying evidence-based demand ensuring ample supplies of drugs are available and affordable. Estimating the LTD would assist decision-making and resource allocation, but many of the clinical and epidemiologic variables are subject to uncertainty. Decision analysis [2] may assist in generating an assessment of the demand for albumin.

## Methods

**Figure 1:**
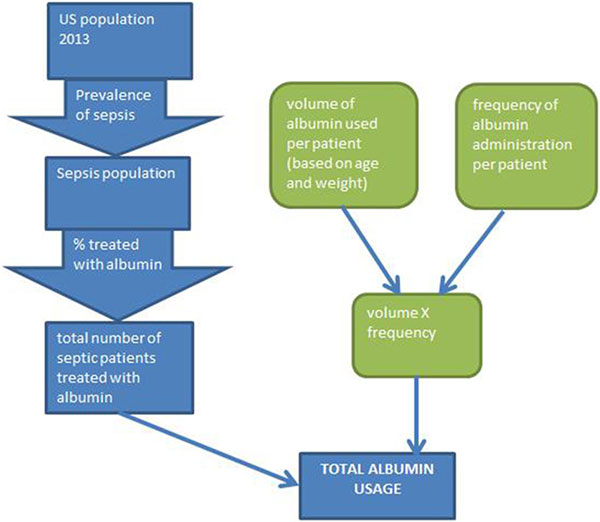
**Variables used to construct the LTD model in sepsis**.

**Figure 2:**
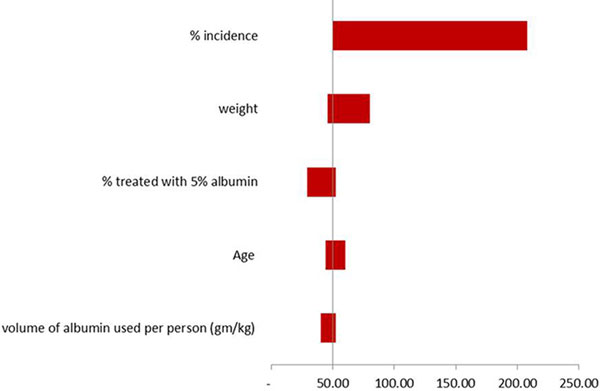
**Relative importance of inputs into the LTD model for sepsis**.

A decision analysis model was constructed using Excel. The model is based on the relationships of the epidemiological and clinical factors shown in the influence diagram (exemplified in Figure 1 for sepsis). Data for the individual factors were obtained from the literature. One-way sensitivity analysis was used to generate Tornado diagrams (exemplified in Figure 2 for albumin use in sepsis) to determine the relative contribution of different factors to the LTD. Probabilistic sensitivity analysis was used to generate a probability distribution and calculate a mean level for the LTD of each indication.

## Results

On average, albumin use was calculated as 104 g per 1,000 inhabitants in severe sepsis, 157 g per 1,000 inhabitants in liver diseases and 61 g per 1,000 inhabitants in CABG. This shows a total LTD of 322 g per 1,000 use of albumin in the US annually.

## Conclusion

Albumin consumption in the USA currently averages 479 g per 1,000 population [3]. Hence, the LTD of these three evidence-based indications represents 67% of current usage. Further work is needed to assess the LTD for albumin in other, less well-defined areas.
